# Research on the influence of digital finance on the economic efficiency of energy industry in the background of artificial intelligence

**DOI:** 10.1038/s41598-023-42309-5

**Published:** 2023-09-11

**Authors:** Qiao He, Ying Xue

**Affiliations:** 1 School of Economics and Management, Xi’an University of Technology, Xi’an, 710000 Shaanxi China; 2 School of Finance, Shanghai Lixin University of Accounting and Finance, Shanghai, 201209 China

**Keywords:** Ecology, Environmental sciences, Environmental social sciences, Solid Earth sciences

## Abstract

China's economic growth has reached a new plateau. It is no longer appropriate to use the old economic growth model, which relied on labor, land resources, mineral resources, and other economic considerations. Under the background of artificial intelligence, high-quality economic development is an inevitable trend. A new financial paradigm called "digital finance" integrates financial services with information technologies. Digital financial technology is thought to be a crucial foundation for fostering high-quality and sustainable economic and social development since it may offer more economic entities reduced cost of capital and more realistic financial service skills than in traditional financial models. In the era of artificial intelligence, how to reasonably release the momentum of digital finance for China's sustained economic growth has become a hot topic of discussion at this stage. This paper studies the impact of digital finance on the economic efficiency of the energy industry in the context of artificial intelligence. Relevant metrics were also calculated. The findings revealed that: The benchmark regression result of digital finance on the efficiency of the green economy was 0.4685 before adding the main restrictions; the benchmark regression result of digital finance on the efficiency of the green economy was 0.2243 after adding the main constraints. As a result, data finance had a favorable impact on the effectiveness of the green economy.

## Introduction

The coordinated growth of the real economy and the digital economy takes the conventional "reverse integration" path, with the financial sector serving as the first example of its transformation and development characteristics in the tertiary sector. Digital finance generally refers to the use of electronic information technology by traditional financial institutions and online businesses to carry out new financial services like payment, project investment, and equity financing. The limitations on time and space between product transactions and financial services have been eliminated by the quick development of digital finance. In the era of artificial intelligence, what is the role of the rise and application of digital finance in the critical period of innovation in promoting strategic planning. Whether it can fill the shortcomings of the traditional financial system and improve the efficiency of urban green economic development and the financial market department's strong support and promotion of innovation and manufacturing, and better support the economy of the energy industry, requires further review and debate. Digital financial technology can provide more accurate Market trend forecast and energy price forecast by processing large-scale data and applying advanced data analysis technology. This will help energy companies make more intelligent decisions, optimize resource allocation, reduce production and operating costs, and thus improve Economic efficiency. The application of digital financial technology can improve the Economic efficiency of the energy industry, improve the efficiency of resource utilization, reduce waste and promote the sustainable development of the energy industry.

## Literature review

Numerous professionals and academics have always focused their research on strategies to increase the effectiveness of the urban green economy. In order to create a model that could be used for urban green economy planning, Liu T enhanced the conventional algorithm and merged the principle of machine learning algorithm. Efficiency indicators for the green economy were evaluated in terms of input, anticipated output, and unexpected production. Comparison and analysis were done on the green efficiency determined using the relaxation value calculation model. The study's findings demonstrated that the model could be used in the design phase of urban green planning and that it had specific effects^1^. China's green economic efficiency and green total factor productivity were assessed and examined by Gao X. Furthermore, the shortcomings of conventional clustering techniques in high-dimensional data clustering were highlighted by outlining the properties of high-dimensional data. A sampling and residual squared-based density peak clustering technique was put forth. The experimental comparison on the data set revealed that in terms of time complexity and clustering outcomes, the modified algorithm outperformed the delayed procedure call approach^2^. Sarcheshmeh M examined the performance of urban green space in terms of social and economic indices in the Mashhad metropolitan region. 15 social questions and 5 economic questions from the research questionnaire were tested and examined using the SPSS22 program. The findings demonstrated that there was no appreciable impact on the management effectiveness of the urban green space sector in the city of Mashhad. From the perspectives of citizens and managers, several features of the social index were rated as desirable^3^. In order to examine the dynamic changes in the economic effectiveness of urban land use in South Korea at the regional level and to determine whether it would be feasible to implement the green belt policy, Yongrok C used the ecological efficiency measurement model. In order to increase the economic benefits of urban land use and execute sustainable green space management, more performance-oriented policy solutions were advocated^4^. These studies do have some impact on increasing the effectiveness of urban green economy and urban planning, but digital finance has received far too little attention. The market for digital finance is quickly taking over with the pace of the new economic system. The city's long-term development would have an effect on how effective the urban green economy is.

There are more research on the direct or indirect effects of digital finance on economic growth than there are on the effect of digital finance on the effectiveness of urban green economies. Based on the database for the growth of digital financial inclusion and the China Family Panel Studies, Xie W investigated the relationship between coastal rural residents' entrepreneurship and the development of China Family Panel Studies (CFPS). The empirical findings indicated that a crucial factor in encouraging rural entrepreneurship was the thorough development of digital financial inclusion. The monetary capital index and the payment index both significantly boosted rural inhabitants' entrepreneurial activity. The study also discovered that the effects of digital financial inclusion on rural residents' entrepreneurship exhibited signs of geographical variation^5^. In the context of economic digitization and the development direction of contemporary financial technology legal supervision, Barykin S determined the function of digital finance in the financial system. By adding new features of digital assets, the digital financial cube might be expanded to match the level of openness of industrial firms in the future Industry 4.0 technological framework^6^. The long-term causal impacts of digital financial inclusion on economic growth in sub-Saharan Africa were investigated by Thaddeus K J. The study made use of quarterly data from 2011 to 2017 and a sample of 22 sub-Saharan African nations. The findings indicated a long-term causal link between digital financial inclusion and economic growth in sub-Saharan Africa, with the causal relationship running one way from economic growth to inclusion in the latter^7^. Rastogi S set out to investigate how unified payment interface affects financial inclusion, economic development, and financial literacy of the underprivileged in India. He discovered that financial literacy was being impacted. Financial stability and trust both served as partial moderators of the significant associations between digital financial inclusion and economic development as well as the significant link between financial literacy and financial inclusion. This fostered financial inclusion and economic growth for the underprivileged in addition to supporting financial literacy^8^. Lin Boqiang uses the non radial direction distance function to build green Economic efficiency indicators that can evaluate cities at prefecture level and above in China under the super efficiency framework, and further empirically studies the impact of economic agglomeration on green Economic efficiency. To solve the endogenous problem caused by reverse causality between economic agglomeration and green Economic efficiency^9^.

The perfect combination of digital technology and financial services has created a new financial service model. With the help of intelligent digital technology, digital finance can provide lower capital cost and faster service mode for the real economy, provide financial services with "high efficiency, convenience and sustainable commercial services" for the energy industry, and complete the unification of objectivity and precision of financial services. This paper discusses the influence of digital finance on the economic efficiency of the energy industry under the background of artificial intelligence, and aims to provide theoretical guidance for the improvement of the green economic efficiency in the energy industry.

## The influence mechanism of digital finance on the economic efficiency of the energy industry

New energy technologies include solar power generation, water energy, wind energy, tidal energy, sea surface temperature difference energy, wave energy, firewood, peat soil, biochemical material energy conversion, geothermal energy, tar sand, etc. At this stage, it is generally recognized that new energy and renewable resources are based on the development trend of new technology application, and gradually change the development and utilization of renewable resources. The traditional fossil energy resources with environmental pollution problems and limited total amount should be replaced by new energy sources that will not be limited by the total amount and the utilization of the recycling system. The key development areas include solar power generation, tidal energy, hydrogen energy and wind energy.

The new energy industry is the exploration, development and utilization of new energy. It uses social methods to achieve effective utilization and popularization, including the whole process of scientific research, industrial utilization, production, manufacturing and operation. It is a high-tech that commercializes solar power generation, wind energy, bioenergy, etc. From the perspective of the characteristics of the industrial chain, the new energy industry is to replace the new industries with strategic status represented by fossil energy, and has extremely important obligations in replacing fossil energy, promoting economic growth, protecting the environment, and building a harmonious society; From the perspective of the whole industry chain, the new energy industry can be divided into energy supply, product research and development, investment and manufacturing, transportation and trading.

The Corona Virus Disease 2019 pandemic has had a major impact on the traditional financial services provided by financial institutions, but it has also accelerated the digital transformation of these services. According to the statistics and analysis of the China Asset Appraisal Association, during the epidemic period, the average service item replacement rate of online banking reached 96%. Despite the epidemic's considerable effects on small and micro businesses and traditional financial "long-tail clients", However, under the background of the intelligent era, the development speed of digital banking is enough to solve the problems of these groups. Through "zero contact" to provide them with low-cost, convenient and fast service projects, especially the contact-free loan has become an important means to help the sustainable development of the energy industry^10^.

**Figure 1:**
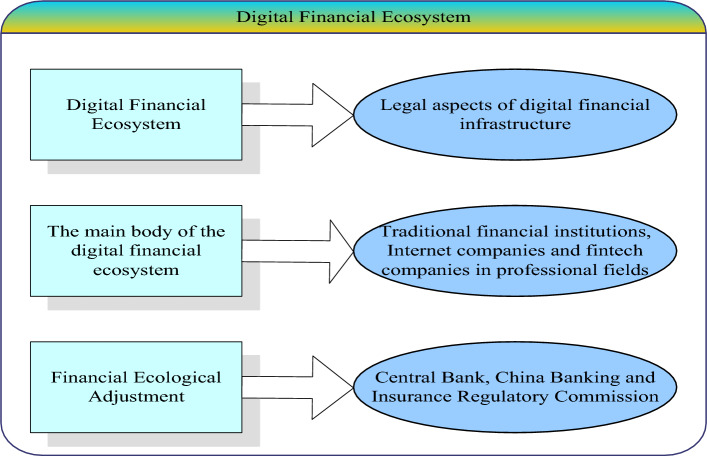
Digital financial ecosystem.

The development of digital finance requires a complete institutional system, and the institutional system of digital finance is the financial ecosystem, which is composed of the main body of the ecosystem and the financial ecological environment. The close combination of the two can produce a regular financial ecosystem with internal logic and self-improvement. Judging from the current overall situation of China's financial institution management system, it has basically formed a large digital financial service ecological chain dominated by banking, Internet banking, non-bank finance, and large and medium-sized financial high-tech companies with electronic payment system, integrity management system, legal norms as infrastructure and institutional guarantee, which is dominated by the "one committee, one bank, two committees and one bureau" supervisory agency^11,12^. A schematic representation of the structure of the digital financial ecosystem is given in Fig. 1.

At this point, a significant trend is the close integration of digital technology with finance. In the era of artificial intelligence, digital technology is playing a unique and important role in modern finance. The following points mostly highlight the benefits of digital finance: Firstly, by increasing financing channels, the threshold for financial services has been lowered; secondly, by greatly reducing service prices, comprehensive financial services have achieved sustainable development; thirdly, the personalized financial services can better meet the various requirements of different users; the fourth is to help reduce information asymmetry and provide new risk management methods^13^.

**Figure 2:**
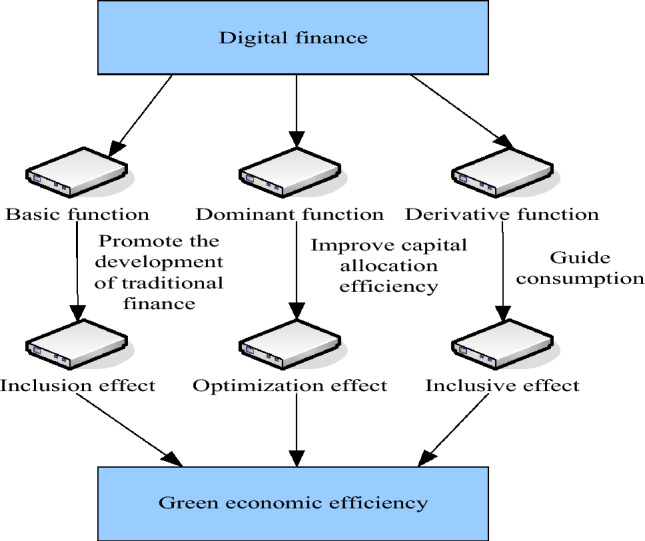
The impact of digital finance on how well urban green development is carried out.

According to different levels of financial functions, digital finance can be divided into three categories: basic functions, leading functions and derivative functions. Figure 2 shows the mechanism of digital finance on the efficiency of urban green development. There are three behavioral paths for the above three functions. The first is digital finance → intermediary services → inclusive utility. Digital finance uses digital information technology to manufacture and expand the role of finance. The network effect of digital technology expands the boundaries of traditional financial services and reduces the service cost of traditional finance. The scale and economic characteristics of digital finance reduce the entry threshold and related costs for innovative enterprises. At the same time, by relying on digital technology, the ability to obtain data and analyze information has been greatly improved and the information asymmetry and the cost of credit intermediary companies have been reduced, and the credit environment has been optimized. After building a three-dimensional credit image based on enterprise big data and cloud technology, sporadic enterprises and start-up companies that are difficult to obtain the support of traditional credit services would obtain a high probability of credit. In order to increase the effectiveness of the urban green economy, the development of digital finance would also help traditional finance change and grow. It would also make full use of the complementary roles that traditional finance and digital finance play in advancing economic growth. Therefore, digital finance will promote the development of traditional finance, and will promote the economic development of the energy industry, and achieve the effect of improving the economic efficiency of the energy industry^14,15^.

The second is digital finance → resource allocation service → upgrade utility. Resource allocation service is the core role of finance and an excellent way to correctly guide use value. On the one hand, the birth of digital finance has promoted competition among financial formats and enhanced the charm of folk capital and the financial system, and improved the efficiency and capability of capital allocation. The use of artificial intelligence and electronic information technology can better match the investment needs and financing needs, reduce the financing pressure of the energy industry, and make the capital used more efficiently and quickly for innovation. On the other hand, the circulation of capital factor commodities has been improved. For a long time, in the factor market, the government department has the dominance and dominance of the vast majority of manufacturing factors, and there may be behaviors such as abuse of power. In addition, the popularity of local protectionism and the emergence of administrative systems have resulted in serious market segmentation. The inconsistency and segmentation of the elements of the sales market make some enterprises, especially state-owned enterprises, lose the driving force of "self-innovation". This harms the development of the urban green economy's efficiency. To provide enough financial factors for the supply-side structure's green development, Digital finance enables the energy industry to overcome regional barriers and enhance the environment for the free flow of capital. Therefore, by enhancing and upgrading the efficiency of regional capital element allocation, data finance can achieve the effect of boosting the efficiency of urban green economy^16^.

The third is digital finance → redistribution of finance → inclusive utility. The rapid development of inclusive finance, on the one hand, helps low-income people get rid of poverty and become rich, which improves the level of per capita consumption and promotes economic transformation and upgrading; on the other hand, with the expansion of the number of netizens and network coverage and the rapid rise of e-commerce and Internet consumer finance, the consumption structure of urban residents has also gradually changed. The demand-side consumption capacity and consumption structure have been upgraded, and the energy industry has increased its demand for high-quality products. This has prompted the energy industry to expand the scope of its technology investment and product development efforts, and to encourage the growth of a local green economy. Therefore, digital financing encourages the energy industry to expand technology investment and product research and development, which has the effect of improving the efficiency of urban green economy^17^.

The energy industry is an indispensable part of economic development. Digital finance provides loans to small and medium-sized energy enterprises to meet the financing needs of small and medium-sized energy enterprises, thus stimulating regional economic growth. However, these small and medium-sized energy enterprises are struggling with financial problems and high financing costs. Only a small number of enterprises can apply for loans from financial institutions through official channels, and other enterprises are under pressure of capital loans. The growth of financial inclusion through digital means has reduced borrowing costs and simplified processes. By providing special loans to such enterprises to help them improve their financing and risk management capabilities, it will help improve their profitability and ultimately improve China's economic growth rate^18,19^.

**Figure 3:**
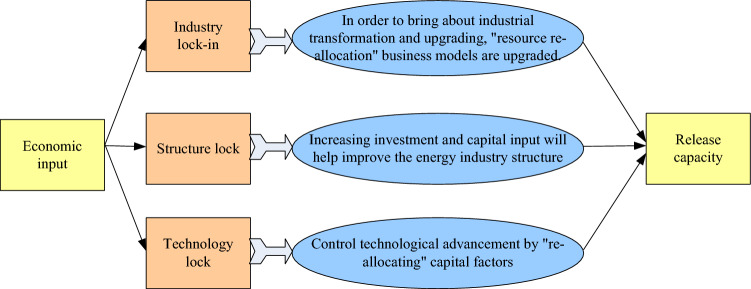
Anti-lock-in support effect structure diagram of microfinance.

If the capital supply cannot keep up, there will be a lock-in effect, and it is imperative to get rid of this inefficient equilibrium state. The basic strategy is to provide specific capital elements for the energy industry, so the assistance of participating banks is essential, and micro loans for small and medium-sized energy industries can help them achieve higher output. Continuous investment in capital and technology will reduce marginal costs, which will have an impact on increasing output and income^20,21^. As shown in Fig. 3, the structure of micro credit's anti lock support effect.

This paper discusses the impact of digital finance on the economic efficiency of the energy industry in the context of artificial intelligence. The calculation formula of some indicators related to the measurement of the economic efficiency of the energy industry is as follows:

1 $${GTFP}_{au}={\sigma }_{0}+{\sigma }_{1}{GTFP}_{a,u-1}+{\sigma }_{2}{df}_{au}+{\sigma }_{3}{df2}_{au}+{\sigma }_{b}{T}_{bau}+{\theta }_{a}+{\varepsilon }_{a}+{\omega }_{au}.$$

$T$-set of control variables; ${GTFP}_{au}$-Green economic efficiency of energy industry; ${df}_{au}$-digital finance; ${df2}_{au}$-square term of digital finance; ${\omega }_{au}$-disturbance term; ${\theta }_{a}$-time fixed effect

2 $${n}_{au}={m}_{au}{\prime}\sigma +\left(1,{m}_{au}{^\prime}\right)\rho 1\left({g}_{au}>\varphi \right)+{\theta }_{a}+{\omega }_{au},$$

${m}_{au}{^\prime}$-a collection of independent variables; ${\mathrm{g}}_{\mathrm{au}}$-threshold variables

3 $${GTFP}_{au}={\sigma }_{0}+{\sigma }_{1}{GTFP}_{a,u-1}+{\sigma }_{2}{df}_{au}+{\sigma }_{3}{df2}_{au}+{\sigma }_{b}{T}_{bau}+{distrk}_{au}+{\theta }_{a}+{\varepsilon }_{a}+{\omega }_{au},$$

4 $$distrk={\sigma }_{0}+{\sigma }_{1}{distrk}_{a,u-1}+{\sigma }_{2}{df}_{au}+{\sigma }_{3}{df2}_{au}+{\sigma }_{b}{T}_{bau}+{\theta }_{a}+{\varepsilon }_{a}+{\omega }_{au},$$

$distrk$-degree of capital misallocation

5 $${\mathrm{lngdp}}_{\mathrm{au}}={\updelta }_{0}+{\updelta }_{1}{\mathrm{lncapital}}_{\mathrm{a},\mathrm{u}}+{\updelta }_{2}{\mathrm{lnlabor}}_{\mathrm{a},\mathrm{u}}+\frac{1}{2}\times {\updelta }_{3}{\left({\mathrm{lncapital}}_{\mathrm{a},\mathrm{u}}\right)}^{2}+\frac{1}{2}\times {\updelta }_{4}{\left({\mathrm{lnlabor}}_{\mathrm{a},\mathrm{u}}\right)}^{2}+{\updelta }_{5}{\mathrm{lncapital}}_{\mathrm{a},\mathrm{u}}\times {\mathrm{lnlabor}}_{\mathrm{a},\mathrm{u}}+{\omega }_{au},$$

${\mathrm{lngdp}}_{\mathrm{au}}$-degree of capital distortion

6 $${MP}_{au}=\left({\delta }_{1}+{\delta }_{3}{lncapital}_{a,u}+{\delta }_{5}{lnlabor}_{a,u}\right)\times \frac{{gdp}_{au}}{{captial}_{au}},$$

${MP}_{au}$-margin of capital

7 $$min\gamma =\frac{1-\frac{1}{K}\sum_{k=1}^{K}\frac{{X}_{k}^{d}}{{d}_{k0}}}{1+\frac{1}{L+1}\left(\sum_{l=1}^{L}\frac{{X}_{l}^{e}}{{e}_{l0}}+\sum_{j=1}^{J}\frac{{X}_{j}^{z}}{{z}_{j0}}\right)},$$

$\mathrm{d}$-$\mathrm{d}$ kinds of inputs; L-L kinds of expected outputs; J-J kinds of undesired outputs; $\upgamma$-green total factor productivity efficiency value.

Restrictions:

8 $$\sum_{v=1}^{V}{m}_{g}{d}_{kg}+{X}_{k}^{d}={d}_{k0},k=\mathrm{1,2},\cdots ,K$$

9 $$\sum_{v=1}^{V}{m}_{g}{e}_{lg}-{X}_{l}^{d}={e}_{l0},l=\mathrm{1,2},\cdots ,L$$

10 $$\sum_{v=1}^{V}{m}_{g}{z}_{jg}+{X}_{j}^{z}={z}_{j0},j=\mathrm{1,2},\cdots ,J.$$

Let the formulas be:

11 $${X}_{k}^{d}\ge 0, {X}_{l}^{d}\ge 0, {X}_{j}^{z}\ge 0, {m}_{g}\ge 0$$

12 $$\sum_{v=1}^{V}{m}_{g}=1$$

13 $${cap}_{au}=\left(1-{\propto }_{a}\right){cap}_{a,u-1}+{F}_{a,u-1},$$

${\mathrm{cap}}_{\mathrm{au}}$-fixed capital stock of the whole society; ${\propto }_{\mathrm{a}}$-capital depreciation rate

14 $${cap}_{a,0}=\frac{{F}_{a,1}}{{o}_{a}+{\propto }_{a}},$$

${\mathrm{cap}}_{\mathrm{a},0}$-cap initial capital stock; ${\mathrm{o}}_{\mathrm{a}}$-cap average annual growth rate.

## Empirical study on the impact of digital finance on economic efficiency of energy industry

**Figure 4:**
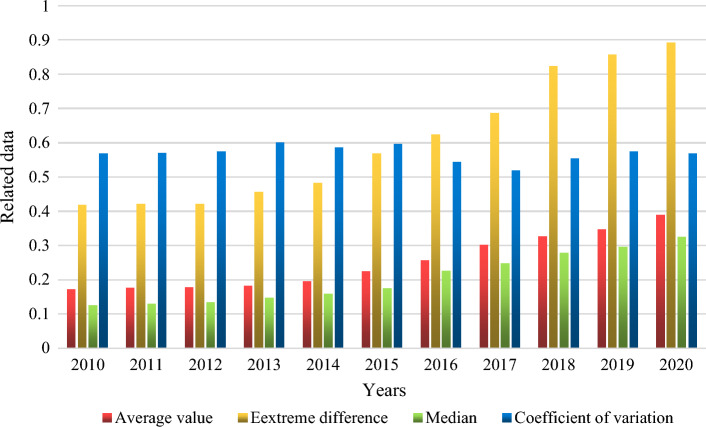
Change index of green economic efficiency development of energy industry in some cities.

In order to explore the impact of digital finance on the economic efficiency of the energy industry in the context of artificial intelligence, we calculated some indicators of the economic efficiency development level of the energy industry^22,23^. Kao (1999) Panel data cointegration test uses the correlation information between individuals to decompose Panel data into inter individual mean and intra individual changes. If the inter individual mean is non-stationary and the residual term is stationary, then the existence of cointegration can be verified. The results are as follows:

As shown in Fig. 4, the change index of green economic efficiency development of energy industry in some cities of China from 2010 to 2020. We selected 20 cities in China for data analysis. The standard deviation is used to measure the Statistical dispersion of a group of data. The larger the standard deviation, the higher the volatility of the data. The average is the average of the green Economic efficiency development index. From the average and median, the average development level of green economic efficiency of these energy industries has increased from 0.1782 in 2012 to 0.3891 in 2020, and the median has also increased from 0.1342 in 2012 to 0.3247 in 2020. Both are rising year by year. From these two indicators, the green economic efficiency level of the energy industry shows a trend of doubling, this also means that the green economy development level of the energy industry has made a qualitative leap. The coefficient of variation did not change significantly from 2010 to 2020, with a value of 0.5687 in 2010 and 0.5682 in 2020. From the perspective of range and coefficient of variation, the range describes the difference between the highest level and the lowest level. In 2012, the range value of green economic efficiency of the energy industry was 0.4213, while in 2020, the range value of green economic efficiency of the energy industry was 0.8925, which also shows an increasing trend year by year. This means that the gap between the development levels of green economy of the energy industry is increasing year by year, while the difference between the extreme values from 2018 to 2020 shows a trend of slowing growth, this also shows that we are also increasing the level of green economy development in economically backward energy industries. It can be seen from the figure that the coefficient of variation of the green economic efficiency of the energy industry fluctuates, but it does not change much, and even shows a downward trend, which also shows that the green development level of the energy industry does not show a development trend of two-level differentiation.

**Figure 5:**
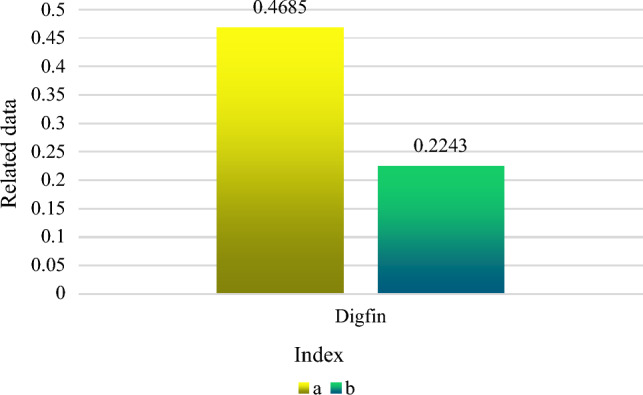
Digital finance and green economy efficiency benchmark regression results.

Regression analysis is conducted with or without control variables to examine the robustness of digital finance on the effectiveness of green economy in the energy industry^24^. The regression results of the efficiency standards of green economy and digital finance in the energy industry are shown in Fig. 5. Where, A represents the result of basic regression without major control factors, and B represents the result of benchmark regression including major control components. It can be seen that before adding the main restrictions, the benchmark regression result of digital finance on the effectiveness of green economy is 0.4685. After the main limiting factors are included, the benchmark regression result of the effectiveness of digital finance on the green economy is 0.2243. Therefore, data finance has a beneficial impact on the effectiveness of the green economy. The green development level of the energy industry does not show a trend of two-stage differentiation, and the benchmark regression results slightly decrease after adding limiting factors. Digital finance will affect the green development level of the energy industry.

The benchmark regression coefficient results of the influence of pertinent variables on green economic efficiency are shown in Table 1. It is clear that the benchmark regression coefficients for improving industrial structure, economic development level, and income from both the public sector and higher education are all positive and pass the 5% significance level test. This demonstrates how investing in financial education, upgrading the industrial structure, and the degree of economic development all help the green economy grow and become more efficient. Despite being positive, the benchmark regression coefficient of environmental legislation on green economic efficiency fails the test of significance. The expense of reducing environmental pollution has perhaps increased, which forces businesses to implement relevant technology advancements. The benchmark regression coefficient for openness to the effectiveness of the green economy is negative, and thus failed the significance threshold test. This may be because the entry of foreign high-tech has raised pressure on environmental governance by bringing about not only economic development but also an industrial chain that produces a lot of pollution and uses a lot of energy.

**Table 1:** Benchmark regression coefficients of related variables’ impact on green economy efficiency.

Variable name	Numerical value	Pass the significance level test
Industrial structural upgrade	0.1456	5%
Environmental regulation	0.0342	No
The level of economic development	0.0637	1%
Openness	− 0.0396	No
Financial education investment	0.1821	5%

Choosing cross-sectional analysis with fixed effects rather than random effects means that there are fixed differences between individuals, and the impact of these differences on variables is constant. This fixed effects model assumes that individual specific factors have a significant impact on the observed variables, and these factors are fixed during the observation period.

**Figure 6:**
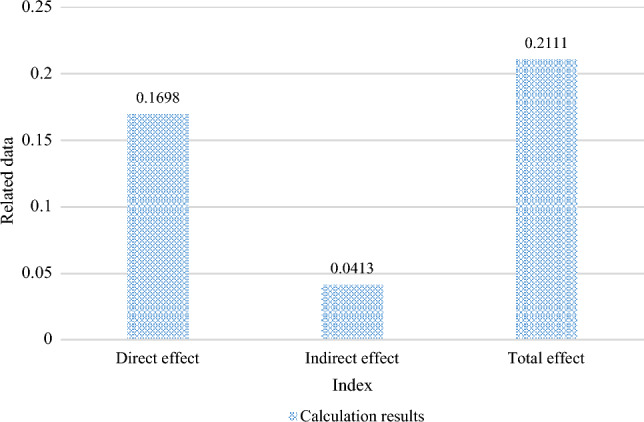
Conduction effect calculation results.

The computation of the conduction effect is shown in Fig. 6. They are digital finance-green economy development efficiency, digital finance-scientific and technical innovation-green economy efficiency, and digital finance-green economy efficiency as a whole. The conduction line of direct effect is digital finance-green economy efficiency. It can be seen that the computed value of the direct relationship between digital finance and green economic efficiency is 0.1698, indicating that the growth of urban green economic efficiency would be directly impacted by the development of digital finance. The calculated indirect effect value is 0.0413, which suggests that digital finance can boost technological innovation to make cities more environmentally friendly by saving energy and lowering consumption and pollution. The level of green economic growth can be raised while industrial upgrading is encouraged. The total effect of digital finance on the effectiveness of green economy in the energy industry is the sum of its direct effect and indirect effect, of which the intermediary effect accounts for 19.56% of the total effect.

**Figure 7:**
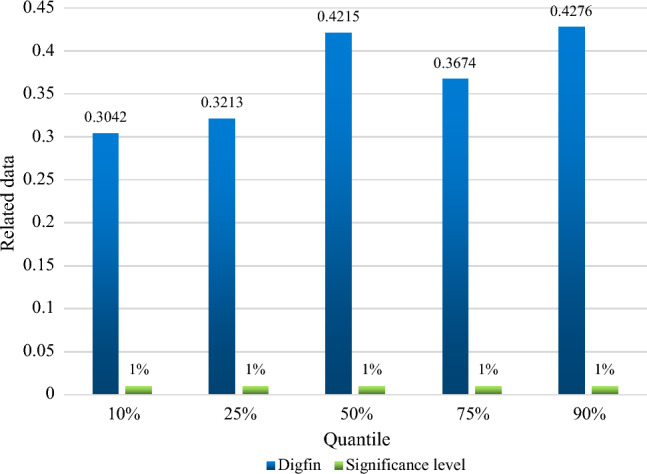
Panel quantile regression results of digital finance and green economy efficiency.

The panel quantile estimation can assess the effect of digital finance on it under each quantile based on the distribution of green economy efficiency levels. The efficiency of the green economy and digital finance are shown in Fig. 7 as the panel quantile regression findings. It is can be seen that for the five quantiles, the estimated coefficient of digital finance climbs as the quantile increases from 0.3042 for the 10% quantile to 0.4276 for the 90% quantile. The increase in the favorable effect is 0.1234, and the significance threshold is 1%. In other words, digital finance has a good effect on the effectiveness of the green economy, and the promotion effect would get stronger as the quantile value rises. This does not help digital finance increase the efficiency of the green economy. However, as the green economy expands and digital infrastructure continues to advance, the beneficial role that digital finance plays in fostering the growth of the green economy would only grow.

In the panel Quantile regression analysis data of digital finance and green Economic efficiency, the estimation coefficient of digital finance is constantly improving, and the significance threshold has always been 1%, so the rise of quantile value will make the promotion of green Economic efficiency stronger.

## Conclusions

This paper analyzes the impact of digital finance on the green economic efficiency of energy industry in the context of artificial intelligence, and evaluates the green economic performance of energy industry in some cities from 2010 to 2020. The empirical research results show that the rapid development of digital finance will significantly improve the efficiency of green economy in the energy industry, and show diversity with the change of city size and industrial development level. Digital finance has the synergistic effect of independent innovation and ecological compensation. Through independent innovation and environmental security management, we can jointly improve the efficiency of green economy. Based on this paper, the following suggestions are put forward: encourage financial institutions, insurance and other traditional finance to transform to digital, use data technology to safeguard the traditional financial system, and accelerate the construction of intelligent facilities in various regions; Give full play to the coordinating role of the financial technology service management system in the introduction of innovation policies, patent applications and other aspects. Accelerate the cooperation between the government and the digital financial platform, and give full play to the aggregation effect of financial markets and policies on independent innovation. Make full use of the ecological compensation effect of digital finance on production units, promote financial innovation through joint development of digital finance, and promote the growth of small and medium-sized enterprises in the upstream and downstream of the green industrial chain and supply chain. The government can formulate policies to encourage energy companies to adopt digital financial technologies, such as blockchain, Big data analysis and artificial intelligence, to improve efficiency and reduce costs. For example, the government can provide tax or subsidy incentives to encourage enterprises to invest in the research and application of digital technology. At the same time, it is necessary to prevent losses caused by excessive economic leverage, so that data finance can better provide energy for the urban real economy.

## Data Availability

Datasets generated and/or analyzed during the current study are available from the corresponding author on request.
